# Bridging biology and technology: the rise of 3D bioprinting advancements in infection research

**DOI:** 10.3389/fbioe.2026.1764653

**Published:** 2026-01-23

**Authors:** Sajad Mohammadi, Wendy W. J. Unger, Aldo Ferrari, Caterina Sanchini, Giancarlo Ruocco, Salvatore D’Alessandro

**Affiliations:** 1 Laboratory of Pediatrics, Department of Pediatrics, Sophia Children’s Hospital, Erasmus University Medical Center (Erasmus MC), Rotterdam, Netherlands; 2 Hylomorph AG, Technopark, Zurich, Switzerland; 3 Center for Life Nano – & Neuro –Science – CLN2S, Italian Institute of Technology (IIT), Rome, Italy

**Keywords:** 3D bioprinting, biomaterials, disease modeling, drug discovery, *in vitro*, multicellular systems

## Abstract

The outbreak of infectious diseases and rapid pathogens’ evolution have highlighted the urgency for developing new therapeutics to protect public health and the economy from massive loss. Drug discovery for infectious diseases involves a multi-stage and multi-disciplinary pipeline, often leading to increased risk and mortality due to the prolonged course. However, advancements in technology have been reshaping the field by offering alternative *in vitro* models—facilitating drug discovery, studying the mechanism of infectious diseases, and developing patient-specific solutions. Recently, 3D bioprinting has been emerging as a revolutionary technology that enables researchers to precisely create custom 3D constructs that mimic human physiology and can be used as either platforms for delivering therapeutics and/or cells locally or *in vitro* tissue models for drug screening. Herein, we shed light on recent advancements in the use of 3D bioprinting technologies to introduce platforms employed for fabricating 3D structures to control and study infectious diseases.

## 1 Introduction

Infectious diseases and epidemics caused by the emergence of new or resistant pathogens have been life-threatening for both humans and livestock. Infection occurs by entering the body and multiplying of microorganisms, including viruses, bacteria, fungi and parasites which can spread from person to person, animal to human, or via a contaminated environment (Baker et al., 2022; Piret and Boivin, 2021). Currently, preventing the spread of the disease is at the forefront of infection management, with rapid development of vaccines and therapeutics playing an indispensable role (Ellwanger et al., 2021; Nii-Trebi, 2017). Drug development for infectious diseases is a complex and multistage procedure which includes screening drug candidates, preclinical studies, clinical trials and regulatory approval, making the process slow and costly (DiMasi et al., 2020). In fact, about 90% of drugs face failure in the clinical trial stages. Using 2D cultures during preclinical tests is one of the contributing factors to this low success rate, as these models lack sufficient relevance to human microenvironments and fail to produce accurate data in terms of drug diffusion, efficacy and safety (Zimmerling and Chen, 2020). Therefore, there is a pressing need to explore novel approaches as alternative for conventional methods that can speed up the drug discovery process, yet mimic *in vivo* human microenvironments.

3D bioprinting has been emerging as a novel technology, capable of orchestrating the three-dimensional structures in a precise and consistent layer-by-layer fashion (Tripathi et al., 2020). These 3D arrangements are composed of living cells, biologically active substances and biomaterial inks which have been widely used for tissue engineering and regenerative medicine (TERM), as well as disease modelling purposes (Yu et al., 2021; Maia et al., 2023; Juraski et al., 2023; Jabbari et al., 2025). 3D bioprinting has offered the capability to create complex and functional 3D models of tissues and organs which enable researchers and clinicians to study the mechanisms of diseases, test new drugs and develop personalized treatments (Maia et al., 2023; Yang et al., 2024a; Kashkooli et al., 2025). To date, a variety of 3D bioprinting techniques such as inkjet-based, laser-assisted and extrusion-based have been developed to engineer constructs that accurately resemble human physiology (Wu et al., 2023; Vanaei et al., 2021). More recently, hybrid technologies that combine different techniques such as volumetric bioprinting, melt extrusion, fused deposition modelling with electrospinning and microfluidic-assisted bioprinting, enhanced by computer-aided design (CAD) fabrication of obtained models from medical images have been coming to the fore and offer precise control over spatiotemporal deposition (Mohammadi and Cidonio, 2023; Koch et al., 2021; Lee et al., 2022; Liu et al., 2022; Mohammadi et al., 2024).

While the application of 3D bioprinting technology in TERM, cancer therapy and biomaterial research has been reported in details elsewhere (D’Alessandro et al., 2024; Bini et al., 2023a; Bini et al., 2023b; Tabriz and Douroumis, 2022), the advancements and impacts of 3D bioprinting in infection studies remain less explored. Herein, we delve into the reasons behind using this novel technology, summarize and report its innovations, aiding infection research and highlight the biomaterial inks explored so far.

## 2 The need for alternative methods in preclinical research

Drug discovery is a lengthy and costly process that conventionally takes 10–15 years from early studies on molecular target or the disease pathways to the final approval of the drug candidate to be used in clinic. This process is even more challenging in infectious diseases, mainly due to the frequent emergence of drug resistant pathogens and therapeutic failure (Si et al., 2022). Essential phases in this process are: 1- Discovery and development, 2- preclinical research, 3- clinical research, 4- FDA review and 5- post-market safety monitoring. All these are essential phases, aimed at identification, optimization and ensuring the safety and effectiveness of a promising compound (Singh et al., 2023; Maranesi et al., 2025). Key steps among these phases are preclinical demonstration of formulation, validation, pharmacokinetics, and pharmacodynamics, to assess whether a compound is likely to cause serious impairment. To this point, animal models have been playing a crucial role in the assessment of toxicology, efficacy and potential side effects of newly developed drugs (MacLeod et al., 2024; Jena et al., 2025; Schaller et al., 2025). However, apart from the high cost of animal research, there have been several reports, suggesting that the animal models might be poor predictors of drug safety and human reactions (Bracken, 2009; Van Norman, 2019). Technology advancements, ethical concerns and the urge for more efficient and predictive preclinical models have led to a substantial transformation in preclinical research, mainly in search for alternative methods for animal experiments (Kiani et al., 2022; Marenzana and Vande Velde, 2015).

### 2.1 Principle of the 3Rs: Replace, reduce and refine

For over 50 years, the principle of the 3Rs has been shaping the frameworks for performing more humane animal studies. This principle aims to maximize the use of non-animal methods, such as *in vitro* techniques and computer models; experiment design aimed at minimizing the number of animals while still obtaining valid and reproducible results, and making use of the appropriate technologies to diminish the pain and suffering of animals. Recently, the FDA has announced its plan to phase out animal testing of monoclonal antibodies, and eventually other drugs, which marks a monumental turn in the drug development process. In this regard, researchers are encouraged to utilize 3D tissue cultures, organ-on-a-chip, and AI models to accelerate the development, standardization, and validation of new substances in preclinical settings, as well as study disease mechanisms. While relevant to infection research, the exploration of organ-on-a-chip and AI models is beyond the scope of this review and are reported in detail elsewhere (Vashishat et al., 2024; Gangwal and Lavecchia, 2025; Rudroff, 2024). Therefore, in the next section we review the application of 3D bioprinting in constructing 3D tissue cultures and its innovations in infection management.

## 3 Application of 3D bioprinting in infection management

3D cell cultures, due to better interaction of cells either with each other and the surrounding extracellular matrix (ECM) offer a more physiologically relevant niche that to a degree mimic *in vivo* conditions (Ca et al., 2022). The bioprinted tissues have shown high levels of similarity to native tissues; therefore hold a great potential for studying different aspects of infection such as barrier functions of tissues, pathogens behavior, and the treatment (Yi et al., 2021). In the following, we focus on the tissues/organs with both high infectious diseases relevance and availability of bioprinted models for studying infection—as certain districts still lack sufficient published work to support a robust discussion.

### 3.1 3D bioprinted constructs for prevention/treatment of wound infection

**FIGURE 1:**
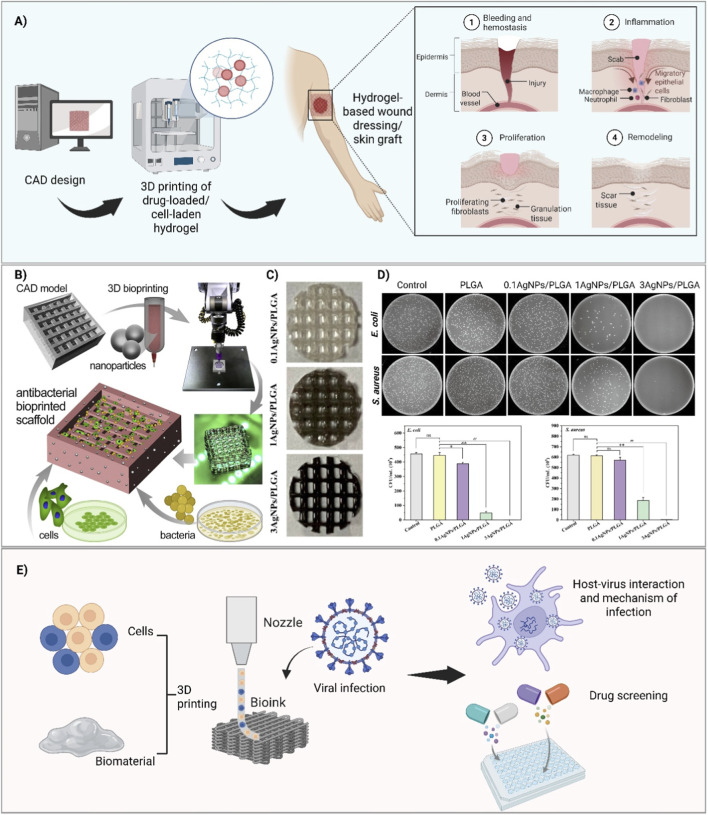
Application of 3D bioprinting in infection management using hydrogels for delivering cells and drugs to the site of interest. **(A)** Schematic representation of wound treatment with 3D bioprinted cell and/or drug-loaded hydrogel dressings. The CAD software enables the precise design of the wound dressing or skin graft, followed by encapsulating cells or required bioactive molecules within the hydrogel; forming the bioink. The bioink will be loaded into printer cartridge to produce the dressing in a layer-by-layer fashion. The dressing can deliver specific cells and therapeutics directly to the wound area, facilitating the wound healing process that composed of hemostasis (stop bleeding), inflammation (immune cells clean up the bacteria and debris), proliferation (migration of fibroblasts and epithelial cells to the wound area to form granulation tissue, blood vessels and new skin on granulation tissue) and tissue remodeling (re-organizing tissue by replacing granulation tissue and forming permanent skin). **(B)** The process of creating and evaluating a 3D printed antimicrobial scaffolds—starting with CAD design, preparing and loading the ink, 3D printing and assessment of antimicrobial properties by co-culturing the scaffold and bacteria. Adopted from (Theus et al., 2022) Creative Common CC BY-NC-ND 4.0 license. **(C)** 3D printed Polylactic-co-glycolic acid (PLGA) scaffolds loaded with silver nanoparticles, using direct ink writing method. **(D)** Assessment of antimicrobial efficacy of the scaffolds against 10^5^ (CFU/mL) *E. coli* and *S. aureus*, depicting significant (p < 0.01) reduction in bacteria viability at when scaffolds were loaded with 3% AgNPs. Adopted from (Chen et al., 2023) Creative Common CC BY license. **(E)** 3D *in vitro* models for studying viral infection. Different cell types can be loaded within hydrogel, resulting in a multi-cellular 3D construct for studying virus-host interaction and mechanism of the infection, as well as testing the efficacy of different drug candidates.

Skin is susceptible to several injuries, including burns, wounds, infections, trauma, and chronic diseases, which disintegrate the structure and damage the function of skin. The healing process of skin, schematically shown in Figure 1A, involves hemostasis, inflammatory reaction, cell proliferation and tissue remodeling—with phagocytic leukocytes, fibroblasts, endothelial and keratinocytes participating in the process (Zhao et al., 2023; Mohammadi et al., 2023). Wound closure is a vital step towards a successful wound healing process which may increase the risk of infection if delayed. Primary closure can be achieved by suturing small wounds, but grafting skin substitutes is required for larger wounds (Wallace et al., 2023). 3D bioprinting has shown fascinating capability to create hierarchical, interconnected and macro/microporous skin scaffolds and wound dressing, made of bioinks, to be aligned with the injured tissue. These constructs not only recapitulate the complexity of human tissue, but also can carry antimicrobial compounds to minimize the risk of starting infection and biofilm formation while accelerating the healing process (Guptha et al., 2024; van Charante et al., 2023). For instance, curcumin and lignin were combined with poly (caprolactone) to prepare an antimicrobial and anti-inflammatory wound dressing. The dressing was 3D printed with semi-solid extrusion method and supplied a sustained release of bioactive agents, resulting in ≈99% prevention of *Staphylococcus aureus* adhesion (Domínguez-Robles et al., 2023).

To be used as skin substitutes, the selected bioinks in 3D bioprinted constructs must meet a number of criteria, including biocompatibility, degradability, printability, and mechanical and biochemical characteristics close to those of native skin. While providing temporary ECM scaffolds for regulating cell behavior, the bioinks eventually determine the success of the printing process. Therefore, it is important that the printed construct maintains its structural integrity upon placement in defect zone, and ensures integration and interaction without triggering a negative immune response (Zhang et al., 2023). The bioinks are usually composed of a hydrogel form of biomaterials to encapsulate different cell types and provide adhesive sites to carry bioactive agents, cell adhesion, proliferation and differentiation (Matai et al., 2020). Printability of bioinks highly depends on rheological properties of the used biomaterials in their hydrogel phase. While hydrogels with higher viscosity have shown higher stiffness, therefore better structural stability of the printed construct, they might cause damage to the encapsulated cells. On the other hand, low viscosity hydrogels are more pleasant to cells, yet make it difficult to create a stable and functional structure (He et al., 2016; Lee et al., 2020). Considering importance of proper viscosity in balancing mechanical properties, Bian et al. suggested an artificial skin substitute using gelatin–hyaluronan hydrogels laden with human dermal fibroblasts, combined with patterned collagen-mimicking nanofibrous films to enhance tensile strength and direct cell behavior. Human keratinocytes were seeded to form the epidermis, resulting in a graded construct with gradient porosity and improved mechanical integrity (Bian et al., 2024).

### 3.2 3D bioprinted antimicrobial scaffolds

Bone is susceptible to severe defects, stemming from various causes such as trauma, tumors, poor prognosis, and congenital conditions. Adhesion of *Staphylococcal* species, specifically *S*. *aureus* is often considered as the main cause of bone infection (Du et al., 2023). Treating infections in the presence of bone defects imposes significant challenges to the orthopedics field. 3D printing of biocompatible antimicrobial scaffolds with appropriate mechanical strength is an enticing approach to overcome concerns of microbial infection, as shown in Figure 1B. To this end, combination of biodegradable materials including polymers, ceramics, graphene, bioglass or metals with antibiotics, antimicrobial peptides or nanoparticles has gained significant attention (Yang et al., 2024b; Xu et al., 2024; Cheng et al., 2021).

**FIGURE 2:**
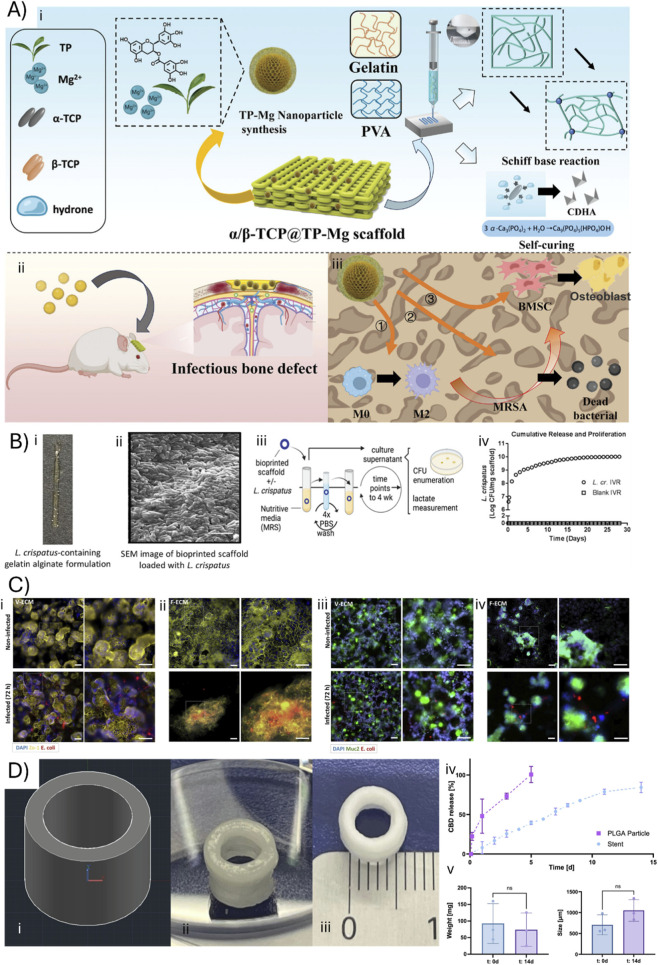
**(A)** (i, ii) The schematic illustrates a self-setting bioactive bone repair scaffold enriched with TP-Mg, designed for the treatment of infectious bone defects. In this system, α-TCP forms a stable mineralized surface through hydration reactions, while collagen gains enhanced flexibility via interaction with Schiff bases. (iii) TP-Mg assists in the healing of infected bone lesions through three distinct mechanisms: (1) eradication of pathogenic bacteria at the infection site; (2) induction of macrophage polarization towards an anti-inflammatory phenotype; and (3) stimulation of osteogenic differentiation in mesenchymal stem cells. Adopted with permission from (Hu et al., 2024). **(B)** (i) Three-dimensional bioprinted scaffolds made from gelatin and alginate, loaded with *Lactobacillus crispatus*, are developed to provide sustained delivery in vaginal applications. The scaffold is produced with extrusion 3D bioprinting approach with different ratios of gelatin to alginate. Scale bar in images is 1 mm. (ii) Additional images present ring-shaped scaffolds, either blank or containing *L. crispatus* at a concentration of 5 × 10^7^ colony-forming units (CFU)/mg. Scale bar is 5 mm. (iii) A schematic outlines the experimental procedure, showing the incubation of 3D printed devices in MRS medium, with washes performed between each timepoint, (iv) followed by enumeration of CFU to assess bacterial viability. Adopted with permission from (Kyser et al., 2023). **(C)** (i, ii) Intestinal cell cultures formed on V-ECM maintained robust barrier integrity after *E. coli* infection, with cells remaining closely joined and continuously expressing the tight junction protein Zo-1. In contrast, those grown on F-ECM displayed significant cell loss and disruption of tight junctions following infection, despite partial preservation of villi. The images depict how the pre-existing mucosal barrier on V-ECM effectively protects against *E. coli* by preventing pathogen adhesion, proliferation, and biofilm formation, while F-ECM-supported tissues show mucin expression only at select villi and experience widespread dissociation of the undifferentiated cell layer in the presence of infection. Fluorescent microscopy of tissues grown on V-ECM and F-ECM, stained for Zo-1 and Muc2 before and after infection. Scale bars, 50 µm. Adopted with permission from (Abdollahi et al., 2025). **(D)** (i) The 3D printed urethral stent was developed starting from its CAD model. (ii) The fabrication involved combining laponite hydrogel with PLGA particles loaded with encapsulated CBD, then using 3D printing to produce the stent shaped specifically to fit the female urinary tract. (iii) Representative images show the stent after printing and crosslinking. (iv) Drug release and structural stability tests were conducted using artificial urine and a simulated urinary tract. (v) A harvested porcine urinary tract was used to implant the stent in the urethra, with daily washes in artificial urine to replicate physiological conditions. The stent maintained its weight over 14 days, indicating sustained structural integrity (mean ± SD; n = 3). Only minimal swelling was detected during the 14-day test period, further confirming that the stent preserved its physical structure throughout the simulation (mean ± SD; n = 3). Adopted with permission from (Eugster et al., 2025).

Blending antimicrobial compounds with scaffold materials in a desired ratio and subsequent fabrication is the most common method to create antimicrobial scaffolds. Bai et al. used melt electrohydrodynamic 3D printing to fabricate a composite antimicrobial scaffold made of PCL/polyethylene glycol/roxithromycin. *In vitro* drug release study demonstrated an initial burst release followed by sustained drug release behavior that was effective against *E. coli* and *S. aureus* (Bai et al., 2020). Moreover, Tan et al. designed 3D-printed calcium phosphate scaffolds integrating MXene and berberine, as shown in Figures 1C–E, employing photothermal stimulation to trigger sustained antibacterial release and effective healing of infected mandibular bone defects *in vivo* (Tan et al., 2024). Expanding on this concept, Zhang et al. introduced a three-phase bionic scaffold created by extrusion 3D bioprinting where antimicrobial, immunomodulatory, and regenerative cues are sequentially delivered, resulting in high bacterial clearance and robust tissue restoration in animal models (Zhang et al., 2024). Moreover, as shown in Figure 2A, Hu et al. developed multifunctional scaffolds loaded with TP-Mg nanoparticles, demonstrating potent local infection control and enhanced bone formation in large infectious bone defects (Hu et al., 2024). These studies collectively illustrate the capability of 3D bioprinting to deliver complex therapeutic strategies for bone infection management, underscoring its potential to synchronize infection eradication with accelerated bone healing in translational research. These developments signal growing opportunities for highly personalized solutions.

The application of 3D printed antimicrobial scaffolds is not limited to treating infection at bone defects site. Over the past decade, antimicrobial hydrogel scaffolds have been developed for regenerative endodontic procedures. Effective disinfection of root canal is a vital step toward dental pulp stem cell tissue regeneration. However, eliminating resistant microorganisms and residual bacteria during decontamination remained challenging. Ortega et al. loaded gelatin biomaterial ink with 150 μg mL^-1^ and 250 μg mL^-1^ benzyldimethyldodecylammonium chloride and printed the 3D construct by an extrusion-based 3D printer, using a 27G nozzle. The 3D printed scaffolds exhibited antimicrobial and antibiofilm activity against endodontic pathogens, including *Enterococcus faecalis*, *Porphyromonas gingivalis*, and *Streptococcus mutans* (Ortega et al., 2025).

### 3.3 3D bioprinted *in vitro* models for respiratory infection studies

The outbreak of COVID-19 pandemic in 2020 has brought viral infections into the spotlight of prevention strategies and treatment. Upon entering the human body, a virus binds to specific receptors on the host cell surface, penetrates and releases its genome (Ciotti et al., 2020; Wu et al., 2020; Hwang et al., 2023). Although the immune system is typically capable of eliminating viruses via various mechanisms such as cytotoxic cells, interferons and antibodies, the viral infection can spread to other organs through blood vessels if the natural defense system is not strong enough (Hwang et al., 2023). The transmission of viruses into organs can further lead to cell death, severe inflammation and impaired organ function (Fenner et al., 1987).

Due to the rapid spread of newly emerging viral infections and high death toll, as seen in the latest pandemic, potential therapeutics must be developed without delay. However, because of complex mechanism of viral infection and difficulties in obtaining sufficient number of human airway epithelial cells, current 2D and animal models have been falling short in fully recapitulate the transmission rate and route, disease mechanism and pathogenesis of such infection. Lee et al. utilized micro-extrusion and inkjet printing to replicate the architecture and function of the lower respiratory tract by 3D bioprinting a multilayered airway structure laden with endothelial cells, ECM and human lung cell-derived epithelium. The model facilitated molecular profiling of tissue-specific markers and SARS-CoV-2 entry mediator and served as a platform to study host transcriptomic responses and the efficacy of antiviral drugs such as remdesivir and molnupiravir (Lee et al., 2024). Similarly, micro-extrusion bioprinting of alginate, gelatin, hyaluronic acid, collagen, and laminin-521 was used to create a 3D lung model composed of endothelial cells, primary fibroblasts, macrophage cells, and respiratory epithelial cells. This multi-cellular model was used to study infection dynamics with influenza A virus and the Sars-Cov2 omicron variant, over the course of 21 days (Berg et al., 2025).

Bioprinted *in vitro* models have demonstrated an enormous potential as a platform for evaluation of viral infection and antiviral drugs. Despite recent advancements, many models have used immortalized cell lines which may not fully represent human tissue behavior. Therefore, the incorporation of human primary cells or induced pluripotent stem cell (iPSC)-derived cells would facilitate the translation of research findings to clinical settings.

### 3.4 Urinary tract

New applications of 3D bioprinting are revolutionizing the management of urinary tract infections and tissue repair. For instance, as shown in Figure 2D Eugster et al have successfully led an experimental study where a extrusion 3D-printed stents with cannabidiol release was produced for localized treatment of urinary tract infections, demonstrating greater efficacy *in vitro* than traditional systemic therapy (Eugster et al., 2025). Furthermore, as shown in Figure 2B, Kyser et al utilized extrusion 3D printing to create urinary catheters tube with probiotic *Lactobacillus rhamnosus* to prevent catheter-associated urinary tract infections (CAUTI) (Kyser et al., 2025). The catheter was tested *in vitro* under artificial urine flow to assess the stability and the biofilm formation. The constructs maintained their shape and mass for 7 days, with stable *L. rhamnosus* biofilm not removed by the artificial urine media flow. The results indicate that printed scaffolds with probiotics may be effective candidates for preventing CAUTI. These applications have shown how 3D bioprinting can support the development of treatment in urinary tract infection.

### 3.5 Female reproductive system

3D bioprinting has emerged as a promising technology for the treatment and study of infections within the female reproductive system. Experimental studies demonstrate the development of innovative extrusion 3D bioprinted scaffolds incorporating *Lactobacillus crispatus* aimed at preventing bacterial vaginal infections leading to bacterial vaginosis (Kyser et al., 2023). *In vitro* studies demonstrated that probiotic-laden scaffolds effectively inhibited pathogenic colonization, showcasing the potential for localized and sustained probiotic delivery. Building upon infection treatment, subsequent work by Utomo et al focused on 3D-printed vaginal devices, loaded with metronidazole for bacterial vaginosis, showing prolonged drug release and therapeutic promise (Utomo et al., 2023). Additionally, efforts to repair tissue damage following infections have been made. In fact, Zheng et al developed a novel bioink combining vaginal extracellular matrix (vECM), GelMA, and silk fibroin. This composite supported stem cell viability and enhanced tissue regeneration in a rabbit vaginal defect model, significantly improving angiogenesis, epithelialization, and muscle function (Zheng et al., 2024). Furthermore, 3D bioprinting allows biological models to be reproduced that would normally be impossible to explore. Thus, Sun et al advanced the modeling of placental infections through a 3D bioprinted placenta-on-a-chip platform, enabling *in vitro* studies of maternal-fetal barrier infections and drug testing (Sun et al., 2025). Collectively, these experimental approaches emphasize the versatility of 3D bioprinting not only in combating infections but also in facilitating tissue regeneration and studying complex infection dynamics. As the field evolves, such technologies hold great promise for personalized and effective therapies in female reproductive health.

### 3.6 Gastrointestinal tract

3D bioprinting has enabled the creation of engineered constructs and models that advance treatment and mechanistic understanding in gastrointestinal infections. Jiang et al presented a multifunctional bilayer scaffold printed via extrusion 3D bioprinting, incorporating apoptotic extracellular vesicles (ApoEVs) and antibacterial coacervates to prevent infection and promote healing in intestinal wound models, showing enhanced epithelial closure and antibacterial efficacy (Jiang et al., 2025). Vera et al developed a gut-on-chip system with 3D bioprinted villi structures and electrodes for real-time monitoring of the intestinal barrier, enabling high-fidelity simulation of microbial infection and drug screening (Vera et al., 2024). Abdollahi et al engineered a biomimetic 3D substrate mimicking villus-crypt architecture, achieving rapid tissue differentiation and improved natural antimicrobial peptide production, leading to reduced pathogenic infection in cultured gut tissues, as shown in Figure 2C (Abdollahi et al., 2025). Torras et al designed crypt-villus 3D constructs using customized digital light processing-based stereolithography (DLP-SLA) bioprinting, enabling direct bacterial infection modeling and immune response studies within *in vitro* intestinal tissues (Torras et al., 2023).

Together, these studies demonstrate how bioprinting enables spatially precise, functional models and scaffolds that both treat and replicate complex gut infection processes, paving the way for personalized tissue therapies and mechanistic research in gastrointestinal disease.

## 4 Conclusion

3D bioprinting presents unique advantages for infection studies, including the ability to create physiologically relevant models, engineer personalized therapeutic constructs, and facilitate the controlled delivery of drugs and bioactive agents. These technologies allow for precise structural design and can mimic the cellular and extracellular environments found in human tissues, improving the relevance of experimental infection models.

Nonetheless, despite significant advances, several limitations are highlighted in the review. The selection and optimization of bioinks is critical: while higher viscosity hydrogels improve mechanical stability, they may compromise cell viability. Low viscosity hydrogels enhance cell preservation but can make maintaining construct integrity difficult. Many *in vitro* bioprinted models still rely on immortalized cell lines, which do not fully replicate native human tissue responses, limiting translational potential. Looking ahead, it is vital to put efforts into ensuring long-term cell viability, proper vascularization and upscaling the manufacturing process. Furthermore, consideration should be given to ensure that the complex interaction between different cell types and organs and cell migration is achieved. To address the inherent limitations of single tissue or organ infection, integrating multi-organ-on-a-chip (multi-OoC) platforms with 3D bioprinting can offer a promising path. In fact, many pathogens impact the human body systemically; meaning they breach the epithelial barriers and interact with the host immune system and distal organs. Combined use of multi-OoC and 3D bioprinting, although is still in its infancy, further enables cross-organ communication that leads to a more precise disease modeling—thereby facilitating drug screening and development of personalized treatments.

Overall, while 3D bioprinting offers transformative possibilities for infection management and research, further refinement in material properties and cellular composition is required to enhance clinical translation and model reliability.
